# Factors Influencing Genomic Prediction Accuracies of Tropical Maize Resistance to Fall Armyworm and Weevils

**DOI:** 10.3390/plants10010029

**Published:** 2020-12-24

**Authors:** Arfang Badji, Lewis Machida, Daniel Bomet Kwemoi, Frank Kumi, Dennis Okii, Natasha Mwila, Symphorien Agbahoungba, Angele Ibanda, Astere Bararyenya, Selma Ndapewa Nghituwamhata, Thomas Odong, Peter Wasswa, Michael Otim, Mildred Ochwo-Ssemakula, Herbert Talwana, Godfrey Asea, Samuel Kyamanywa, Patrick Rubaihayo

**Affiliations:** 1 Department of Agricultural Production, Makerere University, Kampala P.O. Box 7062, Uganda; dennisokii@gmail.com (D.O.); mwilanatasha@yahoo.co.uk (N.M.); angeltanzito@gmail.com (A.I.); barastere@gmail.com (A.B.); snghituwamhata@gmail.com (S.N.N.); thomas.l.odong@gmail.com (T.O.); wasswa@caes.mak.ac.ug (P.W.); mknossemakula@gmail.com (M.O.-S.); haltalwana@gmail.com (H.T.); Skyamanywa@gmail.com (S.K.); prubaihayo@gmail.com (P.R.); 2 Alliance Bioversity-CIAT, Africa-Office, Kampala P.O. Box 24384, Uganda; l.machida@cgiar.org; 3 National Crops Resource Research Institute, Kampala P.O. Box 7084, Uganda; otim_michael@yahoo.com (M.O.); grasea9@gmail.com (G.A.); 4 Department of Crop Science, University of Cape Coast, Cape Coast PMB, Ghana; frankkumifk@gmail.com; 5 Laboratory of Applied Ecology, University of Abomey-Calavi, Cotonou 01BP 526, Benin; agbahoungbasymphorien@gmail.com

**Keywords:** prediction accuracy, mixed linear and Bayesian models, machine learning algorithms, training set size and composition, parametric and nonparametric models

## Abstract

Genomic selection (GS) can accelerate variety improvement when training set (TS) size and its relationship with the breeding set (BS) are optimized for prediction accuracies (PAs) of genomic prediction (GP) models. Sixteen GP algorithms were run on phenotypic best linear unbiased predictors (BLUPs) and estimators (BLUEs) of resistance to both fall armyworm (FAW) and maize weevil (MW) in a tropical maize panel. For MW resistance, 37% of the panel was the TS, and the BS was the remainder, whilst for FAW, random-based training sets (RBTS) and pedigree-based training sets (PBTSs) were designed. PAs achieved with BLUPs varied from 0.66 to 0.82 for MW-resistance traits, and for FAW resistance, 0.694 to 0.714 for RBTS of 37%, and 0.843 to 0.844 for RBTS of 85%, and these were at least two-fold those from BLUEs. For PBTS, FAW resistance PAs were generally higher than those for RBTS, except for one dataset. GP models generally showed similar PAs across individual traits whilst the TS designation was determinant, since a positive correlation (R = 0.92***) between TS size and PAs was observed for RBTS, and for the PBTS, it was negative (R = 0.44**). This study pioneered the use of GS for maize resistance to insect pests in sub-Saharan Africa.

## 1. Introduction

Insect damage on maize plants and stored grains potentially impedes food security in Africa [1,2,3]. The fall armyworm (FAW) and stem borers in the field, and the maize weevils (MWs) in storage facilities, are some of the most devastating insect pests on the continent. These insect pests cause yield losses ranging from 10–90% leading to loss of grain marketability, and consumer health concerns due to the possible contamination of the grain with mycotoxins, such as aflatoxins [3,4,5,6]. In Africa, tremendous efforts were made during the last two decades to build host plant resistance to insect pests in maize through traditional pedigree (phenotypic)-based selection (PS) with substantial desirable results. Several Africa-adapted maize lines were developed and successfully tested for resistance to MW damage on grains [7,8,9,10,11,12]. Some of the success stories are from the International Center for Maize and Wheat Improvement (CIMMYT) of Kenya through the Insect Resistant Maize for Africa project (IRMA) that produced several storage pest and stem borer resistant maize lines [7,8,13,14,15]. On the other hand, the FAW is a new pest on the continent, first reported in 2016 in West and Central African countries [16], from where it spread throughout the African continent [17]. Hence, although efforts to develop FAW resistant varieties are underway at several institutions, including CIMMYT, published reports of FAW resistant varieties are not yet available [18,19].

The complex nature of insect resistance traits makes PS slow and expensive, and thus, difficult to implement, especially for resource-constrained breeding programs [20]. Application of traditional marker-assisted selection (MAS) is hampered by the necessity to first discover resistance-associated genomic regions through genetic linkage and genome-wide association mapping methods, both with several shortcomings, especially for complex traits [21,22,23]. In addition, genetic linkage and genome-wide association mapping studies have seldom been explored in African germplasm [8,24], which further impedes the application of MAS in the development of insect resistance maize germplasm in Africa. In a previous study, we discovered several quantitative trait nucleotides and genes that are putatively associated with FAW and MW resistance, confirming the quantitative nature of these traits, hence the difficulty in improving these traits through MAS [25]. An alternative to both PS and MAS is genomic selection (GS), which uses whole-genome markers to perform genomic prediction (GP) of breeding values of unphenotyped genotypes, from which one can select superior candidate genotypes for crossing to produce hybrids or to advance to the next generation [26]. GS was reported to achieve up to threefold annual genetic gain in maize improvement when compared to MAS, due to a more efficient accounting of trait-associated quantitative trait loci (QTL), faster selection cycles, and lower phenotyping costs [27,28,29,30,31,32,33].

Several statistical and machine learning GP models with various strengths and weaknesses have been developed to adapt to different contexts that are partly influenced by the genetic architecture of traits (number and effect size of QTL, proportions of additive and non-additive genetic effects) and reproductive classes of plants (allogamous vs. autogamous vs. clonally propagated) [34,35,36]. Therefore, to effectively implement GS in crop improvement programs, it is necessary to employ a holistic approach to determine the best GP strategy for particular breeding targets for given crop species [31,37]. Statistical models for GS vary in their prior assumptions and treatment of marker effects [31]. Parametric models focus on parameter estimates rather than prediction, while nonparametric algorithms give priority to prediction and have fewer assumptions [38]. Some parametric methods assume the SNP effects follow a normal distribution with equal variance for all loci, which seems unrealistic in practice.

Representative parametric methods are ridge regression best linear unbiased predictors (RR-BLUP) [39] and genomic BLUP (GBLUP) [40]. GBLUP was the first GP method to be developed, and it replaced the traditional pedigree-based relationship matrix with a genomic information-based matrix to improve prediction accuracies (PAs) [41]. Parametric methods BayesA [26] and weighted Bayesian shrinkage regression (wBSR) [42], on the other hand, consider a prior distribution of effect with a higher probability of moderate to large effects. Regarding parametric models such as BayesB [43] and BayesCπ [44], assumptions are made that consider the effects of some SNPs to be zero. The Bayesian least absolute shrinkage and selection operator (Bayesian LASSO) assumes that the effects of all markers follow a double exponential distribution [45], whilst the Bayesian sparse linear mixed model (BSLMM), a parametric method developed by Zhou et al. [46], combines the hypotheses of both GBLUP and Bayesian methods and achieves higher PAs than BayesCπ and BayesLASSO. Nonparametric or semi-parametric approaches such as random forest and reproducing kernel Hilbert space (RKHS) [47,48] are better suited for accounting for non-additive genetic effects (37,38), in contrast with parametric genomic prediction models [23,38,47,49]. Several studies compared the performances of GP models under different conditions. In a simulation study, Meuwissen et al. [26] found that while GBLUP achieved PAs of up to 73.2%, BayesA and BayesB comparatively provided additional increases of around 9% and 16%, respectively. However, when a population is composed of close relatives and the target traits are controlled by several small effect genes, the different methods perform similarly [50,51,52]. On the contrary, BayesB and BayesCπ are better when dealing with distant relatives and traits affected by a small number of large-effect loci [23]. Kernel methods such as RKHS are robust in predicting non-additive effects and in solving complex multi-environment multi-trait models [53,54]. Compared to the above-mentioned parametric methods, deep learning techniques such as support vector regression (SVR), multilayer perceptron, and convolutional neural networks models performed poorly in some studies [55,56]. However, there are also instances where RKHS outperformed one or several of the parametric methods, for instance, GBLUP, rrBLUP, and Bayesian algorithms, in terms of several traits in several crops including maize [51,57,58,59]. These results were most likely because nonparametric GS models capture more adequately the non-additive genetic components which are an essential characteristic of complex traits [23,37,38] and hence could be good candidate tools for the prediction of FAW and MW-resistance traits which are controlled by both additive and non-additive gene action [21,23,31,41,60]. Therefore, since GP for maize resistance to insect pests such as FAW and MW is not yet well explored, it is pivotal to compare performances of several available prediction algorithms to inform better future GS programs. Therefore, the Genomic Prediction 0.2.6 plugin of the KDCompute 1.5.2. beta (https://kdcompute.igss-africa.org/kdcompute/home), an online database developed by Diversity Array Technologies (DArT, https://www.diversityarrays.com) for the analysis of DArT marker data, presents great interest for this purpose. It hosts a suite of parametric, semiparametric, and nonparametric GP methods that can be run simultaneously on genotype-phenotype datasets.

Additional factors that influence PAs are the different sizes of the training sets (TSs) and breeding sets (BS) and their genetic relationships, the number of markers used to estimate genomic estimated breeding values (GEBV) of lines, the population structure, and the extent of linkage disequilibrium [21,23,31,41,60]. Since phenotyping is the current bottleneck in plant breeding and one of the disadvantages of GP is the requirement of large TSs for high PAs to be achieved, determination of effective TS composition and size is critical for effective implementation of GS in crop improvement programs [21,61,62,63,64]. Additionally, the best TS determination will depend on the genetic architecture and the extent of population structure of the trait targeted for GP [63], two parameters that are substantially variable among plant breeding traits. Another factor that is a determinant of the predictive ability is the kinship between the TS and the BS (63). Several methods are used for TS optimization and these generally fall into two categories—namely, untargeted and targeted approaches. For the untargeted approach, the TS is determined independently of its genomic information, whereas the targeted method considers the genomic relationship between the TS and the BS as a means of maximizing PAs [65]. However, deciding on the best TS selection method is not straightforward and depends on context [66].

Furthermore, in maize, GPs were previously conducted using either genotypic best linear unbiased estimators (BLUEs) [67,68,69] or best linear unbiased predictors (BLUPs) [31,41,70] as means of phenotypic correction [70]. BLUEs are obtained by treating the genotypic effect of a mixed linear model as fixed effects and provide an estimated mean for each individual of a population equal to its true value. On the other hand, BLUPs generated by considering the genotypic factors as random and allowing for the shrinkage of the means towards the population mean [71]. Whether to use BLUPs or BLUEs in GP is debatable. Phenotypic BLUEs allow avoiding double penalization which BLUPs suffer from. With phenotypic BLUPs, this double penalization is, however, compensated through maximization of the correlation between predicted and true line values, while phenotypic BLUEs do not rely on this shrinkage [70]. However, the shrinkage in the BLUP procedure accounts better for outliers and environmental variabilities [72], permitting better estimates of individual genetic effects than BLUEs [71], and therefore, it usually yields more accurate predictions of phenotypic performance [70,72,73]. Furthermore, BLUPs are better in handling unbalanced data, wherein, for example, the number of individuals is not the same in different locations or in the different replications of an experiment [49,70]. On that basis, the current study was conducted to evaluate the efficacies of different parametric, semiparametric, and nonparametric methods from both statistical and machine learning GP models in generating prediction accuracies (PAs) for maize resistance to FAW and MW in a diverse panel using both genotypic BLUEs and BLUPs.

## 2. Material and Methods

### 2.1. Genetic Material and Field Experiments

The panel used in this study consisted of 358 maize lines with diverse genetic and geographic backgrounds, and they were sourced from the National Crop Resources Research Institute (NaCRRI/Namulonge, Uganda), the International Institute for Tropical Agriculture (IITA/Ibadan, Nigeria), and The International Maize and Wheat Improvement Center (CIMMYT/Nairobi, Kenya). The panel consisted of 71 inbred lines developed for various purposes at NaCRRI; 28 and five stem borer (SB)-resistant inbred lines from CIMMYT [6,13,14] and IITA, respectively; 19 storage pest (SP)-resistant inbred lines [7,8]; and a doubled haploid (DH) panel of 235 lines developed at CIMMYT using six parents—three of which were stem borer-resistant, one was a storage pest-resistant inbred line (these were also included in the population), and two were CML elite lines (one, CML132 was included in the panel) (Supplementary Materials Table S1).

The panel was planted and evaluated in three environments, at Mubuku Irrigation Experimental Station in Kasese, western Uganda in 2017 (316 lines) during the second rainy season (2017B) and the National Crop Resources Research Institute (NaCRRI), Namulonge, central Uganda in 2018 (92 lines) and 2019 (252 lines) both during the first rainy seasons (2018A and 2019A, respectively). Detailed information on these locations is presented in Table 1.

Each combination of location and season was considered an environment, resulting in a total of three environments. An augmented experimental design was adopted in all three environments using six checks in 2017B, two in 2018A, and four in 2019A replicated in all the blocks. The experiments in 2017B, 2018A, and 2019A consisted of twelve, five, and ten blocks, respectively, containing the replicated checks and unreplicated lines and the experiment in 2018A was replicated twice.

### 2.2. Genotyping, Quality Control, and Imputation for Genomic Prediction Analyses

Genotyping of the panel and SNP quality were described in our previous study [25]. In brief, maize leaves at the sixth-leaf stage of development were harvested from 341 of the 358 lines of the panel (5–10 plants per line) in 2017B and 2018A (for lines not captured in 2017B). The leaf samples were oven-dried overnight at 36 degrees Celsius and shipped to the Biosciences east and central Africa (BecA) Laboratory of the International Livestock Research Institute (ILRI, Nairobi, Kenya) for DNA extraction and genotyping. Diversity Array Technology (DArT) genotyping facilities (44) were used to successfully identify 34,509 SNPs from 341 of the 358 lines composing the panel; hence, only these lines were considered for the GP analyses. Duplicate SNPs were first removed using the R package DartR (45), leaving 28,919 unique SNPs (DRSNP) distributed across all the 10 chromosomes of the entire maize genome.

The DRSNP dataset was imputed before GP using KDCompute 1.5.2. beta (https://kdcompute.igss-africa.org/kdcompute/home), an online database developed by Diversity Array Technologies (DArT, https://www.diversityarrays.com) for the analysis of DArT marker data. KDCompute uses a suit of imputation methods to impute the SNP dataset and scores the imputation results by calculating simple matching coefficient (SMC). The method with the highest SMC is considered as optimal and used to impute the original genotypic dataset.

### 2.3. FAW and MW Resistance Phenotyping

After germination, plants were left unprotected to allow sufficient natural pressure of fall armyworm (FAW) population to build up. FAW damage scoring in all the three environments was carried out two months after planting when adequate natural FAW infestation levels had manifested, and scoring was based on a visual assessment using a scale of 1 (no or minor leaf damage) to 9 (all leaves highly damaged) [75], illustrated in Figure S1 [18].

Rearing of and bioassays for MW were performed as described in previous experiments carried out at NaCRRI [76,77]. Weevils were reared prior to the MW bioassay to obtain enough insects aged between 0 to 7 days for infestation. During rearing, standard conditions were provided for weevils to ensure proper acclimatization during the experiment. Rearing was carried out by preparing a weevil-maize grain culture of 300–400 unsexed insects and 1.5 kg of grains contained in 3000 cm^3^ plastic jars incubated for 14 days in the laboratory at a temperature of 28 ± 2 °C and relative humidity of 70% ± 5%, to enhance oviposition. The lids of the jars were perforated and a gauze-wire mesh with a pore size smaller than one mm was fitted on each lid to allow proper ventilation while preventing the weevils from escaping.

After harvesting and shelling, grains of each line were bulked across environments. Then, samples of 30 g were weighed from each grain bulk, aiming to produce three replicates per line for the MW bioassay experiment. However, due to the lack of an adequate amount of grains for most of the lines of the panel, only 64, 123, and 132 lines could generate three, two, and one replicates, respectively, and were therefore considered for the MW bioassay experiment. Each of these samples was wrapped in polythene bags and kept at −20 °C for 14 days to eliminate any weevil infestation prior to the start of the experiment. After this disinfestation process, samples were left to thaw and transferred into 250 cm^3^ glass jars and infested with 32 unsexed weevils. After 10-days of incubation to allow oviposition, all dead and living adult insects were removed. One month after infestation (MAI), each sample was removed from its jar, and the grains and the flour were isolated and their weights were recorded. The total number of holes inflicted by the weevils on the grains was counted along with the number of grains affected by such damages. Additionally, the numbers of dead and living weevils were recorded. After these measurements were collected, the grains were returned to their respective jars and all the measurements were repeated at two and three MAI. The collected data were used to infer, for each sample, the cumulative grain weight loss (GWL), the cumulative number of emerged adult weevil progenies (AP), and the final number of damage-affected kernels (AK).

### 2.4. Statistical Analyses of the Phenotypic Data

Both best linear unbiased estimators (BLUEs) and predictors (BLUPs) were generated using the general linear model with only phenotype option of the software Trait Association through Evolution and Linkage (TASSEL) [78] and the *ranef* function of the R package [79] lme4 [80], respectively. The mixed linear model for generating BLUEs (all factors considered as fixed) and BLUPs (all factors considered as random) for MW traits (GWL, AP, AK, NH, and FP) was as follows:

Y= μ+Replication+Genotype+Error

The mixed model for generating BLUEs (all factors considered as fixed) and BLUPs (with all factors considered as random) for FAW damage scores across environments model was:

Y=μ+Location+Block+Genotype+Location:Genotype+Error

where μ in the two equations is the intercept.

### 2.5. Strategies for TS and BS Determination

#### 2.5.1. MW Resistance Traits

Due to inadequate amount of seeds, only 37% (126 out of 341 that had genotypic data) of lines from the panel had phenotypic data on grain weight loss (GWL), adult progeny emergence (AP), and number of affected kernels (AK). Therefore, to estimate GP accuracies for MW resistance, these 126 lines were used as the TS and the remaining 215 lines with only genotypic data constituted the breeding set (BS).

#### 2.5.2. FAW Damage Resistance

The GP analyses for FAW resistance were carried out on the 341 lines of the panel that were genotyped and phenotyped for FAW damage resistance. To determine TS and BS sizes and compositions for the evaluation of maize resistance to FAW damage, two strategies, namely, random-based TS (RBTS) and pedigree-based TS (PBTS), were used.

#### 2.5.3. Random-Based TS Determination

For the RBTS, 126 (37%) lines used for GPs of MW-resistance traits were used as the TS for FAW to predict the GEBVs of the remaining 215 lines first. To build the second TS for FAW, the 215 (63%) lines used earlier as BS were considered as a TS. Then to determine the third and fourth TSs for FAW, random selections of 75 and 85% of the lines in the entire panel were performed through the Excel formula “*=INDEX($A:$A,RANDBETWEEN(1,COUNTA ($A:$A)),1)*” and dragging until the adequate number of lines for each percentage determined above was obtained.

#### 2.5.4. Pedigree-Based TS Determination

The four datasets determined based on the pedigrees of the lines in the panel (PBTS strategy) are presented in Table 2. For the first dataset (FAW.Ped1), the 235 (68.91%) CIMMYT doubled haploid (DH) lines were used as a TS and the remainder (106 lines) as a BS. Regarding the second dataset, the TS and BS were switched to consider the TS in FAW.Ped1 as BS, and BS in FAW.Ped1 as the TS. The third dataset, FAW.Ped3, had a TS composed of the 294 that were neither stem borer (SB) resistant nor storage pest (SP)-resistant lines from CIMMYT, whilst the 28 SB and 19 SP-resistant lines from CIMMYT constituted the BS. In the last dataset, FAW.Ped4, the 235 DH lines, the 28 SB and 19 SP-resistant lines from CIMMYT, and the five SB-resistant lines from IITA amounting to 287 (84.16%) genotypes were considered as the TS and the remaining 54 lines from NaCRRI lines were considered as the BS (Table 2).

### 2.6. Genomic Prediction Algorithms

The GP analyses were performed using the BLUEs and BLUPs of the phenotypes and the 28,919 DRSNPs. Sixteen algorithms available in 10 GP methods were implemented using the Genomic Prediction 0.2.6 plugin of the KDCompute 1.5.2. beta. The 10 methods were directly translated from functions of five R packages designed for GP analyses:

#### 2.6.1. Bayesian Models

Bayesian models have different prior distributions with a general model that can be as follows: $y=1_{n}\mu +Z\mu +\varepsilon$, where *y* is the vector of observations, *Z* is the design matrix for random effects, and *µ* is the vector of random effects [31].

The BLR (Bayesian Linear Regression) algorithms from the BLR R Package [81] are used to fit the Bayesian ridge regression. The marker effects are assumed to have a Gaussian prior distribution with mean 0 and variance σ^2^, where σ^2^ is unknown and assumed to have scaled *x*^2^ distribution. In the KDCompute genomic prediction 0.2.6 plugin, the Gibbs sampler is run with 4000 iterations and 1000 iterations for burn-in period as default parameters.

The Bayesian Generalized Linear Regression (BGLR) package fits various types of parametric and semi-parametric Bayesian regressions. The parametric Bayesian algorithms used from this package rely on different prior distributions that induce different types of shrinkages of the marker effects [82], including: Gaussian (Bayesian ridge regression, BRR [83]), scaled-t (BayesA [26]), double-exponential (Bayesian LASSO, BL [84]), and two component mixtures with a point of mass at zero and a distribution with a slab that can be either Gaussian (BayesC [44]) or scaled-t (BayesB [43]). In the KDCompute genomic prediction 0.2.6 plugin tool, defaults parameters for running the Gibbs sampler were used: 4000 iterations and 1000 iterations for burn-in period.

Reproducing kernel Hilbert space (RKHS) [47,48] is a semiparametric Bayesian method from the BGLR package implemented on the KDCompute genomic prediction 0.2.6 plugin. The RKHS methods employs a kernel function to convert the molecular markers as a between pairs of observations distances, thereby, generating a square matrix that fits in a linear model. This non-linear regression method is expected to capture dominance and epistasis effects more efficiently. This approach can be modelled as:

$$y=W\mu +K_{h}\alpha +\varepsilon ,$$

where *μ* represents the fixed effects vector and ε is a vector of random residuals. The parameters α and ε are assumed to have independent prior distributions $\alpha \sim N(0,K_{{h\sigma }_{\alpha }^{2}})$ and $\varepsilon \sim N(0,I_{\sigma_{\varepsilon }^{2}})$, respectively, and the matrix $K_{h}$ relies on a reproducing kernel function with a smoothing parameter *h*. The parameter *h* measures the genomic distances among genotypes that can be interpreted as a correlation matrix and it controls the rate of decay of the correlation among genotypes [51]. To perform this analysis, the same number of iterations and burn-in parameters as for the other Bayesian methods described above were set on the KDCompute genomic prediction 0.2.6 plugin.

#### 2.6.2. Mixed Models

The Sommer (solving mixed model equations in R) package [85] was used to implement the *mmer* (mixed model equations in R) function on the KDCompute genomic prediction 0.2.6 plugin. The package solves mixed model equations proposed by Henderson [86]. It works incidence matrices and known variance covariance matrices for each random effect using four algorithms: efficient mixed model association (EMMA) [87], average information (AI) [88], expectation maximization (EM) [89], and the default Newton–Raphson (NR) [90].

The model by Sommer can be formulated as [85]: y=Xβ+Zμ+ε with variance $V(y)=V(Z\mu +\varepsilon )=ZGZ^{\prime }+R$ Additionally, the mixed model equations for this model are:

$${\left[\begin{array}{c} X^{\prime }R^{-1}X\;X^{\prime }R^{-1}Z\\ Z^{\prime }R^{-1}X\;Z^{\prime }R^{-1}Z+G^{-1} \end{array}\right]}^{-1}\left[\begin{array}{c} X^{\prime }R^{-1}y\\ Z^{\prime }R^{-1}y \end{array}\right]=\left[\begin{array}{c} \beta \\ \mu \end{array}\right]$$

where $G=K\sigma_{\omega }^{2}$ is the variance covariance matrix of the random effect µ, from a multivariate normal distribution $\mu \sim MVN(0,K\sigma_{\mu }^{2})$, ***K*** is the additive or genomic relationship matrix (**A** or **A_g_**) in the genomics context, ***X*** and ***Z*** are incidence matrices for fixed and random effects, respectively, and ***R*** is the matrix for residuals (here $I\sigma_{e}^{2}$). A mixed model with a single variance component other than the error ($\sigma_{e}^{2}$) can be used to estimate the genetic variance ($\sigma_{\mu }^{2}$) along with genotype BLUPs to exploit the genetic relationships between individuals coded in **K**(**A**). The genomic relationship matrix was constructed according to VanRaden where $K=ZZ^{\prime }/2\sum p_{i}(1-p_{i})$ [91].

The ridge regression best linear unbiased predictor (rrBLUP) packages can either estimate marker effects by ridge regression, or alternatively, BLUPs can be calculated based on an additive relationship matrix or a Gaussian kernel. Additionally, using the rrBLUP package, **the mixed model solution (MMS)** that calculates the maximum-likelihood (ML) or restricted-ML (REML) solutions for mixed models to perform GP [92] was fitted in the KDCompute genomic prediction 0.2.6 plugin.

The mixed models fitted by rrBLUP can be formulated as:

y=Xβ+Zμ+ε, 

where *β* is a vector of fixed effects and *µ* is a vector of random effects with variance $Var[\mu ]=K_{\sigma_{\mu }^{2}}$. The residual variance is $Var[\varepsilon ]=I_{\sigma_{\varepsilon }^{2}}$. This class of mixed models, in which there is a single variance component other than the residual error, has a close relationship with ridge regression (ridge parameter $\lambda =\sigma_{\varepsilon }^{2}/\sigma_{\mu }^{2}$) (https://kdcompute.igss-africa.org/kdcompute/home).

#### 2.6.3. Machine Learning Algorithms

The R package RandomForest that implements Breiman’s random forest algorithm for classification and regression [93] was used on the KDCompute genomic prediction 0.2.6 plugin to fit the function missForest. Random forest is a non-linear machine learning algorithm that uses a two-layer randomization process to build decorrelated bootstrapped trees. As a first randomization layer, it builds multiple trees using a bootstrap sample of the marker data in the training. Then, a second randomization process is carried at the novel nodes to grow final trees. The random forest method selects at each node of each tree, a random subset of variables, and only those variables are used as candidates to find the best split for the node [94]. To predict the breeding value of a line in the TS, predictions over trees for which the given observation was not used to build the tree are averaged [51]. On the KDCompute 1.5.2. beta platform, both options for the *mtry*, square root and regression (*sqrt*(*p*) and *p/3*, respectively, where *p* is number of variables in x), for the classification of the number of variables randomly sampled as candidates at each split were implemented in this study. Additionally, the trees to grow (*ntree*) was set to 10, while *node size* (minimum size of terminal nodes) and *max nodes* (maximum number of terminal nodes trees in the forest can have) were set to 5 and 10, respectively. The 16 methods used in this study and their statistical characteristics are presented in Table 3.

### 2.7. Cross-Validations and PA Estimation

To calculate the predictive accuracies of each of the 17 methods, a cross-validation approach was performed using the data for the TS with 10 folds and five repetitions amounting to 50 replications. The PAs were estimated as the correlation coefficient (R^2^) averaged across the 50 cross-validation replications between the observed phenotypic values and the predicted genomic-estimated breeding values (GEBV) (https://kdcompute.igss-africa.org/kdcompute/plugins).

## 3. Results

### 3.1. Higher PAs Achieved for FAW and MW-Resistance Traits with BLUPs when Compared to BLUEs across GP Algorithms

**Figure 1:**
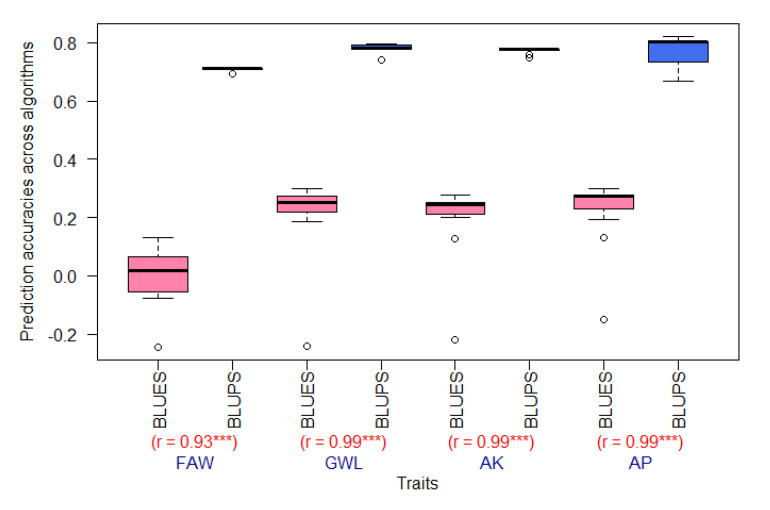
Boxplot of PAs (prediction accuracies) for best linear unbiased estimators (BLUEs) (in pink) and predictors (BLUPs) (in blue) of maize resistance to FAW and MW across prediction models and correlations (r) between BLUEs and BLUPs for each trait. FAW, fall armyworm; GWL, grain weight loss; AP, adult progeny emergence; AK, number of affected kernels. *** significant at *p* < 0.001.

Both genotypic BLUEs and BLUPs for resistance to FAW and MW traits such as AK, AP, and GWL were used in GPs. In general, BLUPs produced better predictions than BLUEs by at least two orders of magnitude in terms of PAs (Figure 1). The PAs realized with BLUEs (Figure S2) varied from −0.246 for FAW (mms_ML) to 0.299 for AP (BayesB), while PAs for BLUPs ranged from 0.668 for AP (mmer_NR) to 0.823 for AP (missForest_Reg). The differences in terms of accuracies between BLUEs and BLUPs were high, despite the highly significant (*p* < 0.001) correlations between BLUEs and BLUPs for each trait ranging from 0.93 for FAW to close to 1 for AP, AK, and GWL (presented in Figure 1); therefore, only results for BLUPs will be presented hereafter.

### 3.2. PAs for MW Resistance Traits Using BLUPs

**Figure 2:**
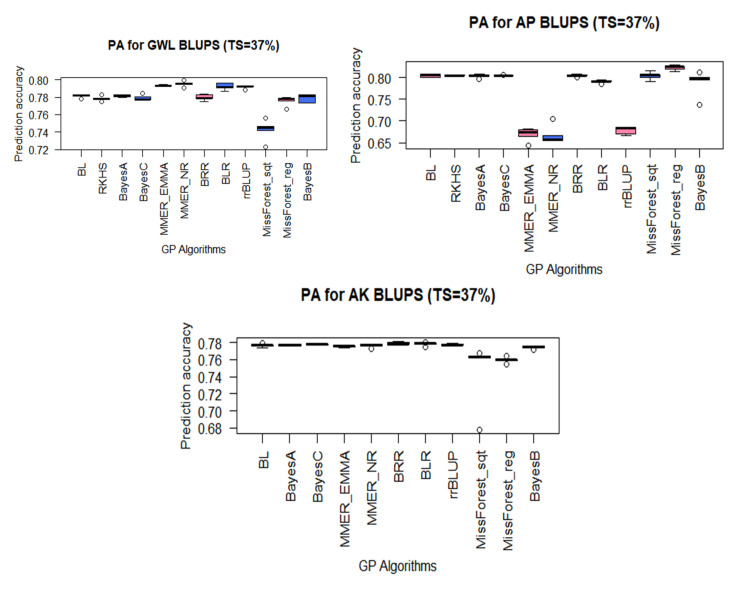
Boxplots of the genomic prediction accuracies of BLUPs for MW-resistance traits: GWL, grain weight loss; AP, adult progeny emergence; AK, number of affected kernels (See Table 3 for GP algorithms).

The PAs were generally high for the tested MW traits, mostly above 0.668 across the 12 GP models that were successfully run on the datasets (Figure 2); however, RKHS failed to work for AK. The highest PAs were achieved for AP with missForest_reg (0.823), followed by BRR (0.805), and RKHS (0.804), whilst mmer_NR algorithm had the lowest prediction accuracy of 0.667 (Figure 2). The PAs achieved for GWL ranged from 0.742 for missForest_Sqt to 0.795 for mmer_NR, while for AK, they varied from 0.749 for missForest_sqrt to 0.779 for BRR (Figure 2). In general, Bayesian models predicted better than both mixed model and machine learning methods, although the differences were small (Figure S3).

### 3.3. PA for FAW Resistance Using BLUPs

**Figure 3:**
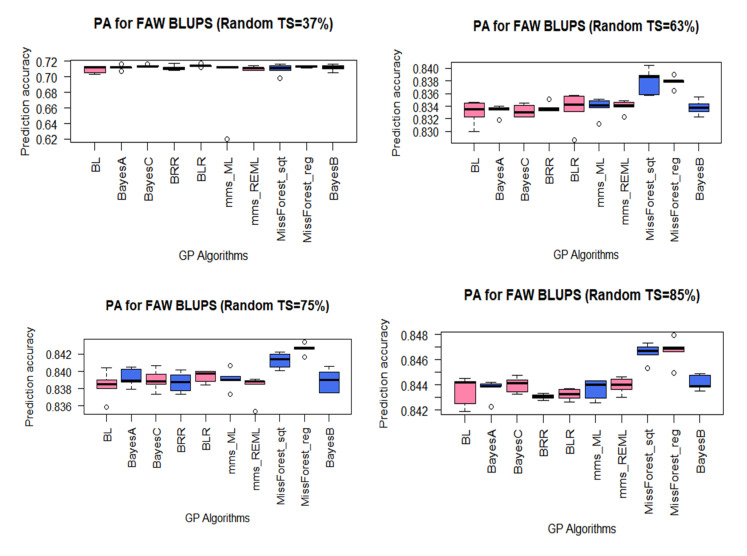
Boxplot of PAs for maize resistance to the fall armyworm (FAW) datasets with the RBTS approach with random selection of 37, 63, 75, and 87% of the entire panel (see Table 3 for GP algorithms).

**Figure 4:**
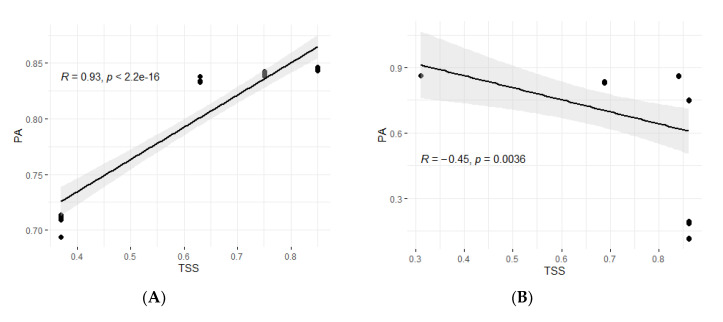
Pearson correlation between training set (TS) sizes and prediction accuracies (PAs) across the 10 genomic prediction algorithms conducted on RBTS (**A**) and PBTS (**B**) datasets for fall armyworm resistance (FAW) resistance.

**Figure 5:**
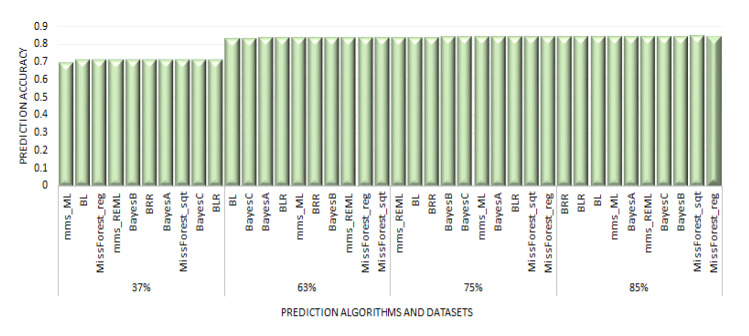
Prediction accuracies for FAW with RBTS across algorithms and training sets with different sizes in percent of the total panel.

The different maize resistance to FAW datasets showed high predictive abilities with 10 of the 16 GP algorithms used in the study. For the RBTS approach, the PAs were lowest with the dataset that had a TS composed of 37% (lowest size) of the panel and highest with the largest TS (85% of the panel). Even with a TS of 37%, the PAs were still high, ranging from 0.694 to 0.714 for mms_ML and BLR methods, respectively (Figure 3). However, it should be noted that with equal TS sizes and same composition (37% of the panel), higher PAs were achieved for MW-resistance traits (GWL, AP, and AK) compared to FAW-resistance ones (Figure S3). The PA for the RBTS of 63% varied from 0.833 for BL method to 0.838 for the missForest_Sqt; thus, there was a small variation among different methods. Similarly, there was minimal variation among GP algorithms on the dataset with a 75% TS whose PAs varied from 0.838 for mms_REML to 0.843 for MissForest_Reg. The same trend was obtained on the dataset with a RBTS of 85% of the panel, with PAs ranging from 0.843 for the BRR model to 0.847 for the missForest_Reg method. Furthermore, there was a high and significant (*p* < 2.2.10^−16^) positive correlation of 0.92 (Figure 4) between the PAs and TS sizes for FAW datasets for the RBTS denoting a steady improvement of the PAs as the TS size increased. However, the PAs for FAW resistance reached a plateau at TS size above 63% of the panel (Figure 5).

**Figure 6:**
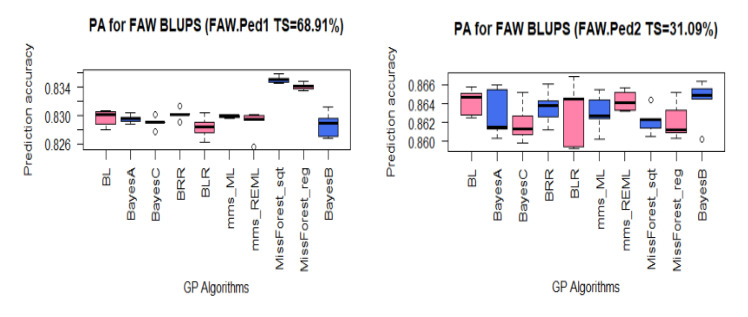

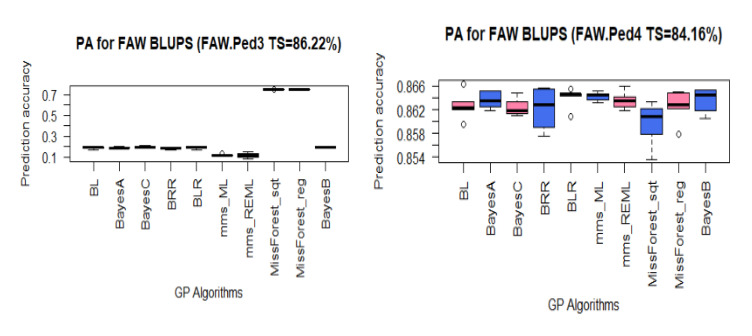
Boxplots of PAs for maize resistance to the fall armyworm (FAW) datasets using the PBTS approach (see Table 2 for the PBTS strategy and Table 3 for GP algorithms).

Although the PAs did not vary much among GP algorithms, especially when the analyses involved larger TS sizes equal or bigger than 63% of the panel, the machine learning methods slightly outperformed other GP algorithms for all the traits, except for the TS of 37% where Bayesian methods such as BLR and BayesC showed a slight advantage over the machine learning methods (Figure S4). The PAs for FAW-resistance datasets with PBTS were generally high, mostly above 0.82 (Figure 6). For the first dataset (FAW.Ped1) with a TS of 68.91% of the panel (see Table 2), the PAs varied between 0.828 for BLR to 0.835 for missForest_Sqt. For FAW.Ped2 (TS = 31.09%), the PAs ranged from 0.862 for BayesC to 0.864 for mms_REML.

For FAW.Ped4, with a TS of 84.16%, PAs varied between 0.860 to 0.864 for missForest_Sqt and mms_ML, respectively. However, for FAW.Ped3 with the largest TS (86.22%), eight of the 10 algorithms achieved low PAs (below 0.20) and only missForest_Reg and missForest_Sqt attained PAs of 0.749 and 0.750, respectively. Thus, the Pearson correlation between the sizes of the PBTS datasets and the predictions accuracies for the 10 GP algorithms revealed a significant (*p* > 0.0036) negative relationship of r = −0.45 (Figure 4).

In the FAW datasets, the PAs were more influenced by the composition of the TS and its genetic relationship with the BS (see Table 2). Using the doubled haploid (DH) lines as TS (FAW.Ped1) and vice-versa (FAW.Ped2) or DH and stem borer (SB) and storage pest (SP)-resistant lines as TS (FAW.Ped4) permitted achieving relatively high PAs from all the 10 algorithms, which when considering the CIMMYT SB and SP-resistant lines as BS and the remainder as a TS (FAW.Ped3), only resulted in machine learning algorithms missForest_reg and missForest_Sqt achieving relatively high PAs. Furthermore, the composition of the TS and its relationship with the BS determined which GP methods achieved the highest Pas; machine learning algorithms worked best on FAW.Ped1 and FAW.Ped3, linear mixed model approaches outperformed Bayesian and machine learning algorithms on FAW.Ped2 and FAW.Ped4, and Bayesian methods ranked either second or third on all datasets (Figure S5). It should be noted that the PBTS strategy generally achieved better PAs than the RBTS irrespective of the size of the TS, except for the FAW.Ped3 dataset (Figure 3 and Figure 6).

## 4. Discussion

Tropical maize germplasm is characterized by rapid linkage disequilibrium (LD) decay with high diversity [95]. These germplasm genetic characteristics make genomic selection (GS) a promising approach to integrate into African breeding programs [96]. However, genomic prediction (GP) models are very diverse and their differential performances depend on crops and trait architectures, besides other parameters such as the size of the training set (TS) and its genetic relationship with the breeding set (BS) [31,37]. Therefore, this study aimed at assessing the feasibility of genomic selection for maize resistance to FAW and MW through estimation of the genomic prediction accuracies achieved by parametric, semiparametric, and nonparametric (machine learning) genomic prediction (GP) algorithms using phenotypic BLUEs and BLUPs, and random and pedigree-based TS determination strategies.

### 4.1. Higher Pas Were Achieved for BLUPs Compared to BLUEs for Both FAW and MW-resistance Traits

With a RBTS of 37% of the panel, which was the smallest and expected to give the worst PAs, PAs were higher (at least two-fold) across both FAW and MW-resistance traits and for all GP models when trait BLUPs were used as phenotypes compared to BLUEs, although there were high Pearson correlations between these two categories of phenotypic data for each trait. In general, BLUPs were reported to have higher predictability than BLUEs owing to better accounting for outliers and environmental variabilities permitted by the shrinkage procedure in BLUPs, which results in more accurate estimates of individual genetic effects [70,71,72,73]. Furthermore, most of the predictive differences between BLUPs and BLUEs might have stemmed from BLUPs being more suitable than BLUEs in fitting data recorded from unbalanced experiments [49,70] as was the case for both FAW damage scores across environments and MW bioassay in this study. Therefore, for all subsequent analyses with higher RBTS sizes and the PBTS strategy for FAW, only BLUPs were focused at in this study and will be further discussed.

### 4.2. High PAs Were Achieved for FAW and MW-Resistance Traits Using Moderately Sized Training Sets

The obtained PAs were high for both MW and FAW-resistance traits even with TS of moderate sizes confirming the potential of genomic selection (GS) in Africa-adapted germplasms [28,29,30,33]. With a TS of 37% of the entire panel, high PAs (above 0.70) for MW-resistance traits, grain weight loss (GWL), adult progeny emergence (AP), the number of affected kernels (AK), and FAW resistance were achieved in agreement with the moderate to high heritability values for these traits as, reported earlier [21,31,41]. These results are significantly important considering that one of the disadvantages of GS is the requirement of large TS which negatively impacts the reduction of phenotyping cost [62,64].

The PAs increased up to above 0.85 in proportion to the increase in TS (RBTS approach) size for FAW resistance which was the only trait phenotyped for all the lines of the panel. It would be interesting to phenotype other lines of the panel that were not evaluated for MW-resistance traits to establish larger TS which may improve the PAs [31,65,97,98]. Very few reports of GP are available for maize resistance to biotic stresses. High PAs were achieved for maize resistance to chlorotic mottle virus (up to 0.95) and maize lethal necrosis (reaching 0.87) in tropical germplasm [67]. However, lower PAs of up to 0.59 were obtained in a study that assessed the predictability of maize resistance to the European corn borer [99] in temperate germplasm. Additionally, Gowda et al. (69) reported moderate PAs (close to 0.60) for maize resistance to a biotic stress, maize lethal necrosis in tropical maize populations.

### 4.3. GP Algorithms Performed Differently on FAW and MW Maize Resistance Traits

In this study, several GP models that included statistical and machine learning algorithms from parametric, semi-parametric, and nonparametric approaches were used to predict FAW and MW-resistance traits. These GP algorithms, as expected, performed differently on the different traits although the predictive variations were generally minimal, especially when large TS were involved, similarly to earlier model benchmarking reports [100,101]. Bayesian models (parametric: BLR and BRR, and semi-parametric: RKHS) performed better on MW traits, GWL, AP, and AK, while nonparametric machine learning algorithms (missForest, here), and to a lesser extent, the linear mixed model (especially in the PBTS approach), achieved the highest PAs on FAW datasets. The differential performances of the different GP algorithms on the insect resistance traits evaluated in this study could be due to differences in the genetic structures (extent of additive vs. non-additive gene action) of the respective traits [23,38,47,49]. Maize resistance to FAW, which was moderately heritable across environments [25], would be expected to be controlled by both additive and non-additive genetic factors, including epistasis [102,103,104], whereas, MW-resistance traits such as GWL, AP, and AK with heritability values above 90% [25] were most likely characterized by a prevalence of additive gene action [105,106] in the current panel.

This supposed genetic architecture difference between FAW and MW-resistance trait could be the reason for non-linear methods such as random forest performing better at predicting FAW resistance, since these are more capable of integrating epistasis in the statistical modelling [27,51]. However, the RKHS algorithm, also a non-linear GP approach known to efficiently handle epistatic genetic relation [51,59], did not successfully run on FAW dataset, although it was among the best models for predicting MW-resistance traits, except BLUPs for the number of affected kernels (AK), for which the RKHS algorithm did not run successfully. In this study, the reasons for some GP algorithms failing to run either on MW or FAW-resistance datasets are unclear, but this could be related to the BLUPS structure of the datasets that failed to run. It should be noted that all the algorithms ran successfully on phenotypic BLUEs datasets with the smallest TS (37% of the panel) being used to compare PAs between BLUPs and BLUEs in this study. However, the two to three-fold predictive ability gain with BLUPs compared to BLUEs would be an incentive to consider BLUPs in future GS activities for maize resistance to MW and FAW. Overall, future GS efforts for maize resistance to MW and FAW are recommended to focus more on Bayesian and machine learning algorithms such as random forest, BayesA, BayesB, BayesC, BRR, and BLR which outperformed mixed linear models for most datasets considered in the current study.

### 4.4. Influences of the Sizes and the Compositions of TS and BS on PAs

Two factors, the relative sizes of the TS and BS (RBTS approach) and their genetic relationship (PBTS approach), influenced the levels of PAs across FAW-resistance datasets, corroborating earlier reports [31,63,65,97,98,107,108]. A net increase in PAs for maize resistance to FAW was realized when the size of the TS was increased from 37% (0.694 to 0.714) to 63% (0.833 to 0.838), similar to earlier reports on wheat yield [109]. This increase was followed by a slight gain in predictability at 75% (0.837 to 0.843) and 85% (0.843 to 0.847), and thus, the PAs plateaued when TS sizes above 63% were considered in this study as reported earlier in other studies [21,64,109,110,111]. Thus, future GS programs for maize resistance to FAW could be designed around TS composed of a minimum of 60% of the entire breeding germplasm to achieve high genetic gains. These results were further supported by the highly significant (*p* > 2.2.10^−16^) positive correlation (R = 0.92) between TS size and PAs. Similarly, positive correlations between the number of lines in the TS and the PAs, and plateau for the PAs were also reported by Edwards et al. [109].

The composition of the TS and its relationship with the BS are determinant factors for the genomic predictability of complex traits [63,112,113,114]. In the current study, using the PBTS approach, these two parameters were more important than the size of the TS since higher PAs were achieved in FAW.Ped2 (0.862 to 0.864) with a TS of 31.09% compared to all other FAW PBTS datasets, including FAW.Ped3 (0.114 to 0.750), with the largest TS of 66.22%. In fact, FAW.Ped3 achieved the lowest PAs among all the PBTS FAW datasets. These results were further illustrated by the significantly (*p* < 0.0036) negative correlation (R = −0.45) between the sizes of the PBTS and the achieved PAs.

However, it is not very clear why the predictions for the BS FAW.Ped3 (47 CIMMYT SB and SP-resistant lines) and the TS (DH, IITA SB, and NaCRRI lines) led to lower PAs for FAW.Ped3. A possible explanation could be that these two sets were distantly related since only two and one CIMMYT SB and SP-resistant lines, respectively, were used as parents to develop the DH lines. Spindel et al. [111] argued that high PAs can be achieved with small-sized TS when lines in the TS and the BS are closely related, since such TS would sample the full genetic diversity of the population. However, the more distantly related the TS and the BS are, the larger the required TS size to reach high PAs [111]. Using the CIMMYT SB and SP-resistant lines as a TS would most likely lead to lower PAs since such a TS would be additionally disadvantaged by its small size (47 lines). The DH lines in the current study are involved as a TS in most of the best performing GP datasets evaluated in the current study (both in the RBTS and PBTS approaches) and as unique lines in the BS of the best performing pedigree-based BS (FAW.Ped2). This DH population could be of interest in future breeding activities targeted at improving insect resistance in maize [23,115,116,117] and potentially useful for GS of complex traits with low to moderate heritability [118].

## 5. Conclusions

This study assessed prediction accuracies of genomic-estimated breeding values for fall armyworm (FAW) and maize weevil (MW)-resistance traits in a diverse Africa-adapted maize panel using several parametric, semi-parametric, and non-parametric genomic prediction models. Prediction accuracies for maize resistance to FAW and MW traits were relatively high, even with a moderate training set size. For FAW resistance, although the prediction accuracies were positively correlated with the size of the training set, the composition and the relationship of the training set with the breeding set were more influential in predicting line performance. Additionally, TS determination-related parameters were more important than the type of genomic prediction models in predicting FAW and MW-resistance traits. However, Bayesian models on MW-resistance traits and machine learning models on FAW damage resistance outperformed mixed linear models in almost all the datasets used in this study. Therefore, future genomic selection programs for maize resistance to insect pests such as FAW and MW in Africa should put more effort into designing effective training sets and use selected Bayesian and machine learning GP algorithms to improve genetic gains, shorten breeding cycles, and accelerate variety release. Such programs could greatly benefit from using the genetically diverse maize panel used in this study as a base population, since it consists of lines adapted to several African agro-ecologies.

## Figures and Tables

**Table 1:** Geographical, climatic, and soil characteristics of the planting locations [74].

Locations	Geographical Coordinates	Altitude above Sea Level	Minimum Rainfall	Soil Characteristics
Kasese	0°16′10″ N 30°6′9″ E	1330 m asl	1000 mm	Sandy loam soils with a pH of 5.68
Namulonge	0°31′30″ N 32°36′54″ E	1160 m asl	1300 mm	Oxisols with a pH of 5.8

**Table 2:** Compositions of the pedigree-based test sets (TSs) for fall armyworm (FAW) datasets.

FAW Datasets	FAW.Ped1	FAW.Ped2	FAW.Ped3	FAW.Ped4
TS composition	235 DH CIMMYT lines	106 Non-DH lines	294 Non-CIMMYT SB and SP resistant lines	287 DH and CIMMYT and IITA SB and SP lines
TS/Panel (%)	68.91	31.09	86.22	84.16

DH = doubled haploid; FAW, fall armyworm; FAW.Ped1 to 4, FAW datasets 1–4 with TS based on pedigree information of the lines in the panel; SB, stem borer; SP, storage pest; TS, training set; CIMMYT, International Center for Maize and Wheat Improvement; IITA, International Institute for Tropical Agriculture.

**Table 3:** Genomic prediction methods used for the analysis of the different traits and datasets.

	GP Algorithms	Abbreviations	Method Type
1	Sommer with Average Information (AI)	mmer_AI	Parametric/Mixed model
2	Sommer with Expectation Maximization (EM)	mmer_EM	Parametric/Mixed model
3	Sommer with Efficient Mixed Model Association (EMMA)	mmer_EMMA	Parametric/Mixed model
4	Sommer with default Newton-Raphson (NR)	mmer-NR	Parametric/Mixed model
5	Ridge-regression Best linear unbiased Predictor	rrBLUP	Parametric/Mixed model
6	Mixed Model solution with Maximum Likelihood (ML)	mms_ML	Parametric/Mixed model
7	Mixed Model solution with Restricted Maximum Likelihood (REML)	mms_REML	Parametric/Mixed model
8	BayesB	BayesB	Parametric/Bayesian
9	BayesA	BayesA	Parametric/Bayesian
10	BayesC	BayesC	Parametric/Bayesian
11	Bayesian least absolute shrinkage and selection operator (LASSO)	BL	Parametric/Bayesian
12	Bayesian Ridge Regression	BRR	Parametric/Bayesian
13	Bayesian Linear Regression	BLR	Parametric/Bayesian
14	Reproducible kernel Hilbert space	RKHS	Semi-parametric/Bayesian
15	Random Forest with Square root	missForest_Sqt	Nonparametric/Machine Learning
16	Random Forest with Regression	missForest_Reg	Nonparametric/Machine Learning

## Supplementary Materials

The following are available online at https://www.mdpi.com/2223-7747/10/1/29/s1, Figure S1: Rating of maize plants based on foliar damage by FAW, Figure S2: Boxplot of PA for best linear unbiased estimators (BLUEs) of maize resistance to the fall armyworm (FAW) and maize weevil (MW) with identical training set size (37%) and compositions, Figure S3: Comparisons of genomic prediction accuracies of the three best algorithms for best linear unbiased predictors (BLUPs) of maize weevil resistance traits: number of affected kernels (AK), adult progeny emergence (AP), and grain weight loss (GWL) vs., fall armyworm resistance dataset with identical TS, Figure S4: Genomic prediction accuracies of the three best algorithms for each fall armyworm resistance BLUPs datasets with RBTS of 37, 62, 75, and 85% of the entire dataset, Figure S5: Genomic prediction accuracies of the three best algorithms for each fall armyworm resistance BLUPs datasets with PBTS, Table S1: Descriptions of parents and crosses that constituted the doubled-haploid population.
